# Methyl 4-nitro­benzoate

**DOI:** 10.1107/S160053680904745X

**Published:** 2009-11-14

**Authors:** Hao Wu, Min-Hao Xie, Pei Zou, Ya-Ling Liu, Yong-Jun He

**Affiliations:** a Jiangsu Institute of Nuclear Medicine, Wuxi 214063, People’s Republic of China

## Abstract

In the mol­ecule of the title compound, C_8_H_7_NO_4_, the nitro group is approximately coplanar with the benzene ring [dihedral angle = 0.6 (1)°], while the dihedral angle between the methoxy­carbonyl group and the benzene ring is 8.8 (1)°. In the crystal structure, weak inter­molecular aromatic C—H⋯O_carbox­yl_ and C—H⋯O_nitro_ hydrogen-bonding inter­actions are present.

## Related literature

For related literature on benzoates, see: Zhang (1992 ▶); Zhang *et al.* (1990 ▶); Zhang *et al.* (1995 ▶).
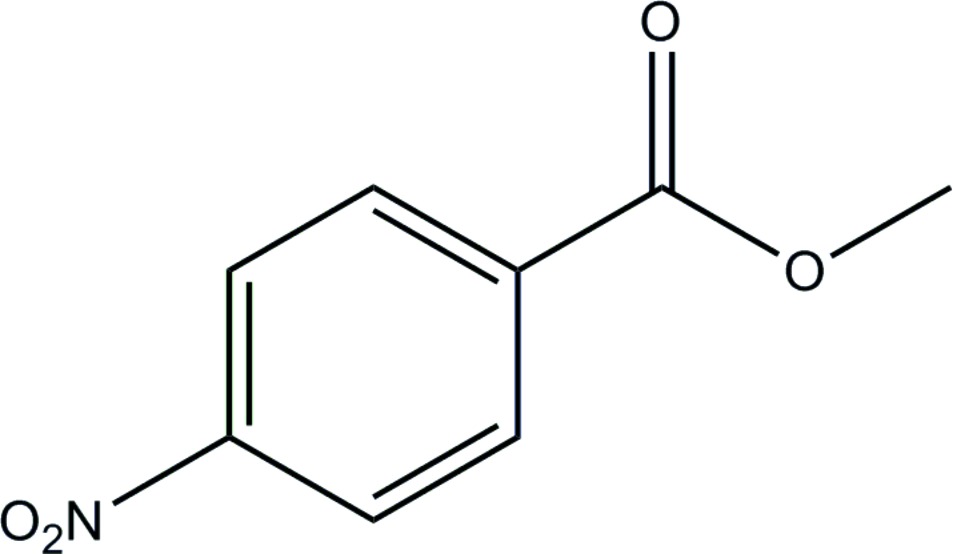


## Experimental

### 

#### Crystal data

C_8_H_7_NO_4_
*M* *_r_* = 181.15
Monoclinic, 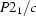

*a* = 7.109 (3) Å
*b* = 17.092 (6) Å
*c* = 7.193 (3) Å
β = 116.292 (4)°
*V* = 783.6 (5) Å^3^
*Z* = 4
Mo *K*α radiation
μ = 0.13 mm^−1^
*T* = 93 K
0.43 × 0.40 × 0.10 mm

#### Data collection

Rigaku SPIDER CCD-detector diffractometer
Absorption correction: ψ scan (North *et al.*, 1968 ▶) *T*_min_ = 0.948, *T*_max_ = 0.988
6176 measured reflections
1787 independent reflections
1445 reflections with *I* > 2σ(*I*)
*R*_int_ = 0.023

#### Refinement

*R*[*F* ^2^ > 2σ(*F* ^2^)] = 0.034
*wR*(*F* ^2^) = 0.080
*S* = 1.00
1787 reflections
119 parameters
H-atom parameters constrained
Δρ_max_ = 0.31 e Å^−3^
Δρ_min_ = −0.18 e Å^−3^

### 

Data collection: *RAPID-AUTO* (Rigaku, 2004 ▶); cell refinement: *RAPID-AUTO*; data reduction: *RAPID-AUTO*; program(s) used to solve structure: *SHELXS97* (Sheldrick, 2008 ▶); program(s) used to refine structure: *SHELXL97* (Sheldrick, 2008 ▶); molecular graphics: *SHELXTL* (Sheldrick, 2008 ▶); software used to prepare material for publication: *SHELXTL*.

## Supplementary Material

Crystal structure: contains datablocks I, global. DOI: 10.1107/S160053680904745X/zs2018sup1.cif

Structure factors: contains datablocks I. DOI: 10.1107/S160053680904745X/zs2018Isup2.hkl

Additional supplementary materials:  crystallographic information; 3D view; checkCIF report

## Figures and Tables

**Table 1:** Hydrogen-bond geometry (Å, °)

*D*—H⋯*A*	*D*—H	H⋯*A*	*D*⋯*A*	*D*—H⋯*A*
C2—H2⋯O2^i^	0.95	2.59	3.384 (2)	141
C5—H5⋯O4^ii^	0.95	2.58	3.378 (2)	142

Symmetry codes: (i) 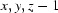; (ii) 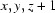.

**Fig. 1.**
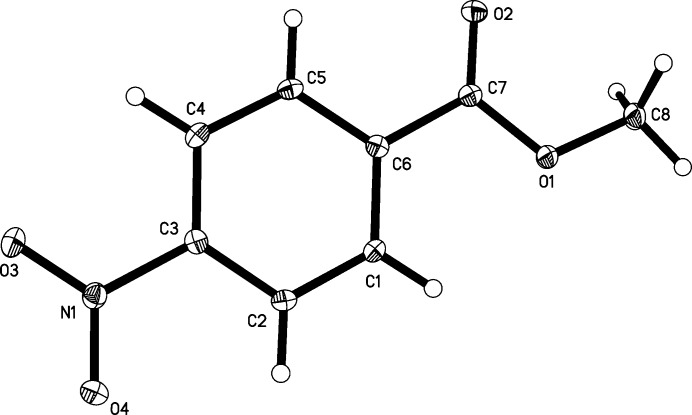
Atom numbering scheme for the title compound (I) with the displacement ellipsoids drawn at the 30% probability level.

## supplementary crystallographic information

### Comment

Benzoates are important intermediates in the chemistry of pigments and pharmaceuticals, which are used worldwide (Zhang, 1992; Zhang *et al.*, 1990; Zhang *et al.*, 1995). We report here the crystal structure of methyl 4-nitrobenzoate, C_8_H_7_NO_4_ (I). In the structure of the title compound (Fig. 1) the bond lengths and angles are within expected ranges. The nitro substituent group is nearly coplanar with the benzene ring [dihedral angle, 0.6 (1)°], while the methoxycarbonyl group forms a dihedral angle of 8.8 (1)° with the benzene ring. In the crystal structure, adjacent molecules are linked by weak intermolecular aromatic C—H···O_carboxyl_ and O_nitro_ hydrogen bonds (Table 1).

### Experimental

4-Nitrobenzoic acid (5.0 g, 30 mmol) was dissolved in hot methanol (10 ml), then six drops of concentrated sulfuric acid were added. The mixture was stirred at 353 K for 4 h, poured into ice water and stirred for 3 min. After filtering, washing with water and drying in vacuum, a white powder was then obtained (yield: 73%). The crude product was purified by recrystallization from methanol (yield: 51%). Colourless plate-shaped crystals [m.p. 369 (2) K] were obtained after several days, by slow evaporation of a 1:1 (*v*/*v*) methanol-water solution. .

### Refinement

Positional parameters of all H atoms were calculated geometrically and were allowed to ride on the C atoms to which they are bonded, with C_aryl_—H = 0.95 Å, and C_methyl_—H = 0.98 Å, and with *U*_iso_(H) = 1.2*U*_eq_(C)

### Crystal data

**Table:**

C_8_H_7_NO_4_	*F*(000) = 376
*M**_r_* = 181.15	*D*_x_ = 1.536 Mg m^−^^3^
Monoclinic, *P*2_1_/*c*	Melting point: 369(2) K
Hall symbol: -P 2ybc	Mo *K*α radiation, λ = 0.71073 Å
*a* = 7.109 (3) Å	Cell parameters from 2257 reflections
*b* = 17.092 (6) Å	θ = 3.2–27.4°
*c* = 7.193 (3) Å	µ = 0.13 mm^−^^1^
β = 116.292 (4)°	*T* = 93 K
*V* = 783.6 (5) Å^3^	Plate, colorless
*Z* = 4	0.43 × 0.40 × 0.10 mm

### Data collection

**Table:**

Rigaku SPIDER CCD-detector diffractometer	1787 independent reflections
Radiation source: rotating anode	1445 reflections with *I* > 2σ(*I*)
graphite	*R*_int_ = 0.023
ω scans	θ_max_ = 27.5°, θ_min_ = 3.2°
Absorption correction: ψ scan (North *et al.*, 1968)	*h* = −9→9
*T*_min_ = 0.948, *T*_max_ = 0.988	*k* = −20→22
6176 measured reflections	*l* = −9→9

### Refinement

**Table:**

Refinement on *F*^2^	Primary atom site location: structure-invariant direct methods
Least-squares matrix: full	Secondary atom site location: difference Fourier map
*R*[*F*^2^ > 2σ(*F*^2^)] = 0.034	Hydrogen site location: inferred from neighbouring sites
*wR*(*F*^2^) = 0.080	H-atom parameters constrained
*S* = 1.00	*w* = 1/[σ^2^(*F*_o_^2^) + (0.0303*P*)^2^ + 0.336*P*] where *P* = (*F*_o_^2^ + 2*F*_c_^2^)/3
1787 reflections	(Δ/σ)_max_ < 0.001
119 parameters	Δρ_max_ = 0.31 e Å^−^^3^
0 restraints	Δρ_min_ = −0.18 e Å^−^^3^

### Special details

**Table:**

Geometry. All e.s.d.'s (except the e.s.d. in the dihedral angle between two l.s. planes) are estimated using the full covariance matrix. The cell e.s.d.'s are taken into account individually in the estimation of e.s.d.'s in distances, angles and torsion angles; correlations between e.s.d.'s in cell parameters are only used when they are defined by crystal symmetry. An approximate (isotropic) treatment of cell e.s.d.'s is used for estimating e.s.d.'s involving l.s. planes.
Refinement. Refinement of *F*^2^ against ALL reflections. The weighted *R*-factor *wR* and goodness of fit *S* are based on *F*^2^, conventional *R*-factors *R* are based on *F*, with *F* set to zero for negative *F*^2^. The threshold expression of *F*^2^ > σ(*F*^2^) is used only for calculating *R*-factors(gt) *etc*. and is not relevant to the choice of reflections for refinement. *R*-factors based on *F*^2^ are statistically about twice as large as those based on *F*, and *R*- factors based on ALL data will be even larger.

### Fractional atomic coordinates and isotropic or equivalent isotropic displacement parameters (Å^2^)

**Table:**

	*x*	*y*	*z*	*U*_iso_*/*U*_eq_	
O1	0.35100 (15)	0.66883 (5)	0.67509 (14)	0.0205 (2)	
O2	0.30933 (15)	0.59510 (5)	0.91414 (14)	0.0216 (2)	
O3	0.14128 (16)	0.28185 (5)	0.23700 (16)	0.0265 (2)	
O4	0.14076 (17)	0.36321 (6)	0.00524 (15)	0.0275 (2)	
N1	0.15702 (17)	0.34819 (6)	0.17881 (17)	0.0185 (2)	
C1	0.25319 (19)	0.54972 (7)	0.39808 (19)	0.0155 (3)	
H1	0.2638	0.6016	0.3563	0.019*	
C2	0.21513 (19)	0.48826 (7)	0.2601 (2)	0.0160 (3)	
H2	0.2007	0.4971	0.1240	0.019*	
C3	0.19872 (19)	0.41347 (7)	0.3264 (2)	0.0155 (3)	
C4	0.21927 (19)	0.39748 (7)	0.5240 (2)	0.0167 (3)	
H4	0.2065	0.3456	0.5643	0.020*	
C5	0.25907 (19)	0.45958 (7)	0.6606 (2)	0.0160 (3)	
H5	0.2749	0.4503	0.7970	0.019*	
C6	0.27592 (18)	0.53560 (7)	0.59852 (19)	0.0149 (3)	
C7	0.31372 (19)	0.60132 (7)	0.7484 (2)	0.0162 (3)	
C8	0.3760 (2)	0.73724 (8)	0.8036 (2)	0.0239 (3)	
H8A	0.2532	0.7425	0.8310	0.029*	
H8B	0.3890	0.7840	0.7313	0.029*	
H8C	0.5025	0.7314	0.9351	0.029*	

### Atomic displacement parameters (Å^2^)

**Table:**

	*U*^11^	*U*^22^	*U*^33^	*U*^12^	*U*^13^	*U*^23^
O1	0.0302 (5)	0.0155 (5)	0.0190 (5)	−0.0039 (4)	0.0139 (4)	−0.0029 (4)
O2	0.0259 (5)	0.0241 (5)	0.0167 (5)	−0.0026 (4)	0.0111 (4)	−0.0005 (4)
O3	0.0390 (6)	0.0150 (5)	0.0290 (6)	−0.0025 (4)	0.0182 (5)	0.0006 (4)
O4	0.0437 (6)	0.0225 (5)	0.0202 (5)	−0.0009 (5)	0.0177 (5)	−0.0016 (4)
N1	0.0196 (5)	0.0163 (5)	0.0211 (6)	0.0005 (4)	0.0102 (5)	0.0002 (4)
C1	0.0151 (6)	0.0144 (6)	0.0172 (7)	−0.0002 (5)	0.0075 (5)	0.0022 (5)
C2	0.0152 (6)	0.0189 (6)	0.0142 (6)	0.0007 (5)	0.0067 (5)	0.0024 (5)
C3	0.0132 (6)	0.0160 (6)	0.0172 (6)	0.0011 (5)	0.0066 (5)	−0.0008 (5)
C4	0.0152 (6)	0.0153 (6)	0.0204 (7)	0.0008 (5)	0.0088 (5)	0.0033 (5)
C5	0.0143 (6)	0.0196 (6)	0.0150 (6)	0.0012 (5)	0.0071 (5)	0.0036 (5)
C6	0.0115 (5)	0.0175 (6)	0.0152 (6)	0.0003 (5)	0.0055 (5)	−0.0002 (5)
C7	0.0134 (6)	0.0181 (6)	0.0162 (6)	0.0005 (5)	0.0059 (5)	0.0017 (5)
C8	0.0339 (8)	0.0176 (6)	0.0243 (7)	−0.0055 (6)	0.0167 (6)	−0.0060 (5)

### Geometric parameters (Å, °)

**Table:**

O1—C7	1.3429 (15)	C2—H2	0.9500
O1—C8	1.4517 (15)	C3—C4	1.3901 (18)
O2—C7	1.2111 (16)	C4—C5	1.3880 (18)
O3—N1	1.2308 (14)	C4—H4	0.9500
O4—N1	1.2290 (15)	C5—C6	1.3962 (18)
N1—C3	1.4770 (16)	C5—H5	0.9500
C1—C2	1.3872 (18)	C6—C7	1.4965 (18)
C1—C6	1.3988 (18)	C8—H8A	0.9800
C1—H1	0.9500	C8—H8B	0.9800
C2—C3	1.3873 (17)	C8—H8C	0.9800
			
C7—O1—C8	115.59 (10)	C4—C5—C6	120.30 (12)
O4—N1—O3	123.79 (11)	C4—C5—H5	119.8
O4—N1—C3	118.11 (10)	C6—C5—H5	119.8
O3—N1—C3	118.10 (11)	C5—C6—C1	120.22 (12)
C2—C1—C6	120.25 (12)	C5—C6—C7	118.78 (11)
C2—C1—H1	119.9	C1—C6—C7	120.98 (11)
C6—C1—H1	119.9	O2—C7—O1	123.89 (12)
C1—C2—C3	118.10 (12)	O2—C7—C6	124.65 (11)
C1—C2—H2	121.0	O1—C7—C6	111.46 (11)
C3—C2—H2	121.0	O1—C8—H8A	109.5
C2—C3—C4	123.11 (12)	O1—C8—H8B	109.5
C2—C3—N1	118.00 (11)	H8A—C8—H8B	109.5
C4—C3—N1	118.89 (11)	O1—C8—H8C	109.5
C5—C4—C3	118.01 (12)	H8A—C8—H8C	109.5
C5—C4—H4	121.0	H8B—C8—H8C	109.5
C3—C4—H4	121.0		
			
C6—C1—C2—C3	0.59 (18)	C4—C5—C6—C1	−0.12 (18)
C1—C2—C3—C4	−0.22 (18)	C4—C5—C6—C7	178.53 (11)
C1—C2—C3—N1	179.66 (11)	C2—C1—C6—C5	−0.43 (18)
O4—N1—C3—C2	0.62 (17)	C2—C1—C6—C7	−179.05 (11)
O3—N1—C3—C2	−179.62 (11)	C8—O1—C7—O2	−3.17 (18)
O4—N1—C3—C4	−179.49 (12)	C8—O1—C7—C6	176.11 (10)
O3—N1—C3—C4	0.27 (17)	C5—C6—C7—O2	−8.15 (19)
C2—C3—C4—C5	−0.31 (18)	C1—C6—C7—O2	170.48 (12)
N1—C3—C4—C5	179.80 (11)	C5—C6—C7—O1	172.58 (11)
C3—C4—C5—C6	0.48 (18)	C1—C6—C7—O1	−8.78 (16)

### Hydrogen-bond geometry (Å, °)

**Table:**

*D*—H···*A*	*D*—H	H···*A*	*D*···*A*	*D*—H···*A*
C1—H1···O1	0.95	2.39	2.7149 (19)	100
C2—H2···O2^i^	0.95	2.59	3.384 (2)	141
C5—H5···O4^ii^	0.95	2.58	3.378 (2)	142

Symmetry codes: (i) *x*, *y*, *z*−1; (ii) *x*, *y*, *z*+1.
